# Reliable Measures of Sarcopenia in Cirrhosis. Comment on: “The Relationship of Obesity, Nutritional Status and Muscle Wasting in Patients Assessed for Liver Transplantation, Nutrients 2019, 11, 2097”

**DOI:** 10.3390/nu12030875

**Published:** 2020-03-24

**Authors:** Maryam Ebadi, Carlos Moctezuma-Velazquez, Rahima A. Bhanji, Aldo J. Montano-Loza

**Affiliations:** Division of Gastroenterology & Liver Unit, University of Alberta Hospital, Edmonton, AB T6G 2X8, Canada; ebadi@ualberta.ca (M.E.); moctezum@ualberta.ca (C.M.-V.); rbhanji@ualberta.ca (R.A.B.)

We read with interest the manuscript by Vidot et al. [1], investigating the relationship between nutritional status and muscle wasting (sarcopenia) in cirrhosis. In this study, the liver-specific subjective global assessment (SGA), which includes assessment of muscle wasting and subcutaneous fat loss [2], is shown to be insensitive for detecting muscle wasting in patients evaluated for liver transplantation (LT) [1], thus highlighting the need for reliable tools for the detection of muscle wasting and subcutaneous fat loss in clinical practice.

Although the present study contributes to evolving knowledge of sarcopenia and its complications in cirrhosis, some notes of caution are in order. Muscle wasting has been defined using sex-specific cut-offs for psoas muscle in patients undergoing elective resection for colorectal carcinoma [3]. The applicability of these sex-specific psoas muscle cut-offs is debatable in cirrhosis and may explain the high rate of sarcopenia; 97% of the male patients, constituting 70% of the study population, were identified as sarcopenic.

Psoas muscle measurement is rapid and detects sarcopenia better than individual anthropometric measurements; however, it has an asymmetrical shape, and the total area of both right and left psoas muscles accounts for approximately 10% to 13% [4,5] of the total muscle area at the third lumbar vertebra (L3) level. Of note, psoas muscle has limited capacity to recognize sarcopenic patients with higher waitlist mortality risk in cirrhosis [4]. Applying the validated definition of sarcopenia in North American patients with cirrhosis, which looks at the total skeletal muscle index (SMI) measured at L3 [6], we have previously demonstrated weak concordance between sarcopenia and malnutrition, as determined by SGA. We also found sarcopenia and not SGA predicts adverse clinical outcomes in cirrhosis. The concordance between malnutrition and sarcopenia was even weaker in overweight/obese patients [7]. Using SMI cut-offs, we found sarcopenia in 38% of patients with cirrhosis (46% in males, 25% in females).

The authors investigated potential predictors of sarcopenia and reported lower levels of testosterone in male and female patients with muscle wasting in comparison to their counterparts with normal muscle mass. The univariate analysis, however showed, every 1 nmol/L increase in testosterone level to be associated with a 20% increased risk of sarcopenia on the LT waiting list. This paradoxical association may be related to misclassification of patients using inappropriate modalities and cut-points. Lower testosterone levels were detected in sarcopenic male patients with cirrhosis [8], and treatment with testosterone was shown to improve muscle mass [9]. Lastly, lack of association between sarcopenia and wait list mortality might in part be due to misclassification of sarcopenia. Regardless of these limitations, the results of this study emphasize the importance of appropriate body composition assessment concurrent with nutritional assessments as part of the LT evaluation program.
